# Proteomic discovery in sickle cell disease: Elevated neurogranin levels in children with sickle cell disease

**DOI:** 10.1002/prca.202100003

**Published:** 2021-05-24

**Authors:** Eboni I. Lance, Lisa M. Faulcon, Zongming Fu, Jun Yang, Donna Whyte-Stewart, John J. Strouse, Emily Barron-Casella, Kimberly Jones, Jennifer E. Van Eyk, James F. Casella, Allen D. Everett

**Affiliations:** 1 Department of Neurodevelopmental Medicine, Kennedy Krieger Institute, Baltimore, Maryland, USA; 2 Department of Neurology, Johns Hopkins University School of Medicine, Baltimore, Maryland, USA; 3 Food and Drug Administration, Silver Spring, Maryland, USA; 4 Division of Pediatric Hematology, Department of Pediatrics, Johns Hopkins University School of Medicine, Baltimore, Maryland, USA; 5 Division of Pediatric Cardiology, Department of Pediatrics, Johns Hopkins University School of Medicine, Baltimore, Maryland, USA; 6 Division of Hematology, Department of Medicine, Duke University School of Medicine, Durham, North Carolina; 7 Division of Cardiology, Department of Internal Medicine, Johns Hopkins University School of Medicine, Baltimore, Maryland, USA; 8 The Smidt Heart Institute, Cedars-Sinai Medical Center, Los Angeles, California, USA

**Keywords:** neurogranin, sickle cell disease, silent cerebral infarction, stroke

## Abstract

**Purpose:**

Sickle cell disease (SCD) is an inherited hemoglobinopathy that causes stroke and silent cerebral infarct (SCI). Our aim was to identify markers of brain injury in SCD.

**Experimental Design:**

Plasma proteomes were analyzed using a sequential separation approach of hemoglobin (Hb) and top abundant plasma protein depletion, followed by reverse phase separation of intact proteins, trypsin digestion, and tandem mass spectrometry. We compared plasma proteomes of children with SCD with and without SCI in the Silent Cerebral Infarct Multi-Center Clinical Trial (SIT Trial) to age-matched, healthy non-SCD controls.

**Results:**

From the SCD group, 1172 proteins were identified. Twenty-five percent (289/1172) were solely in the SCI group. Twenty-five proteins with enriched expression in the human brain were identified in the SCD group. Neurogranin (NRGN) was the most abundant brain-enriched protein in plasma of children with SCD. Using a NRGN sandwich immunoassay and SIT Trial samples, median NRGN levels were higher at study entry in children with SCD (0.28 ng/mL, *N* = 100) compared to control participants (0.12 ng/mL, *N* = 25, *p* < 0.0004).

**Conclusions and Clinical Relevance:**

NRGN levels are elevated in children with SCD. NRGN and other brain-enriched plasma proteins identified in plasma of children with SCD may provide biochemical evidence of neurological injury.

## 1 | INTRODUCTION

Sickle cell disease (SCD) is an inherited disorder of red blood cells that can have widespread systemic effects, including hemorrhagic or ischemic stroke and silent cerebral infarcts (SCI) [1,2]. SCI is defined as ischemic lesions at least 3 mm in diameter visible on T2-weighted magnetic resonance imaging (MRI) that are not associated with a focal neurologic deficit in the same vascular distribution [3–6]. SCI is associated with decreased neurocognitive function [7,8], and increased risk for new or enlarging SCI or stroke [9,10]. Surveillance MRI for SCI is costly and not done routinely at many institutions. The ability to detect children with SCD who are at risk for SCI remains limited, as no laboratory test for SCI exists. As a result, many individuals with SCD and SCI either remain undiagnosed or are diagnosed after they have SCI-related neurocognitive impairments [11]. With recent trials showing successful treatments for decreasing SCI recurrence and stroke, additional methods are needed for diagnosis, prognostication, and assessment of treatment response of SCI and other neurological complications in SCD [12–16].

Proteomic techniques have the potential to identify proteins associated with brain injury in children with SCD [17]. We hypothesized that children with SCD would have circulating brain-derived proteins in plasma that could be used as surrogate markers for subclinical brain injury and provide insight into the pathophysiology of the disease and that these markers may be elevated in children with SCD and SCI in comparison to children with SCD without SCI, or children with SCD when compared to control participants. In this study, LC–MS based proteomics was used to discover potential brain injury associated proteins, and then develop an immunoassay for detection of one of the brain-enriched proteins in plasma from children with SCD. For verification, neurogranin (NRGN), a neuron-specific signaling protein and one of the brain proteins identified in plasma samples from participants with SCD screened for the SIT Trial, was explored further using longitudinal plasma samples collected from participants in the SIT Trial.

## 2 | MATERIALS AND METHODS

### 2.1 | Study population

Plasma used in the discovery and verification SCD cohorts were obtained from participants enrolled in the IRB approved Silent Cerebral Infarct Multi-Center Clinical Trial (SIT) (ClinicalTrials.gov identifier NCT00072761), a multi-center, international clinical study, and stored in a biologic repository, as well as samples from participants with SCI and SCD who declined randomization in the SIT Trial, SCI negative participants with SCD who did not qualify for randomization for the SIT Trial, and healthy control participants without SCD [5]. Control participants were matched by group characteristics, specifically age, gender, and race. Children with SCD were screened for the SIT Trial with blood samples and MRI obtained at enrollment, followed by randomization of patients with SCI to either monthly transfusion or standard of care (observation). The plasma samples used for the proteomics discovery analyses were screening samples from two groups: children enrolled in the SIT Trial with SCD (*n* = 15) and healthy age-matched children with no SCD (*n* = 6), including three children who had sickle cell trait. Samples from children with SCD were divided into two groups: those with SCI (*n* = 7) and those without SCI (*n* = 8) matched for age, hemoglobin (Hb) and white blood cell counts (WBC).

**FIGURE 1:**
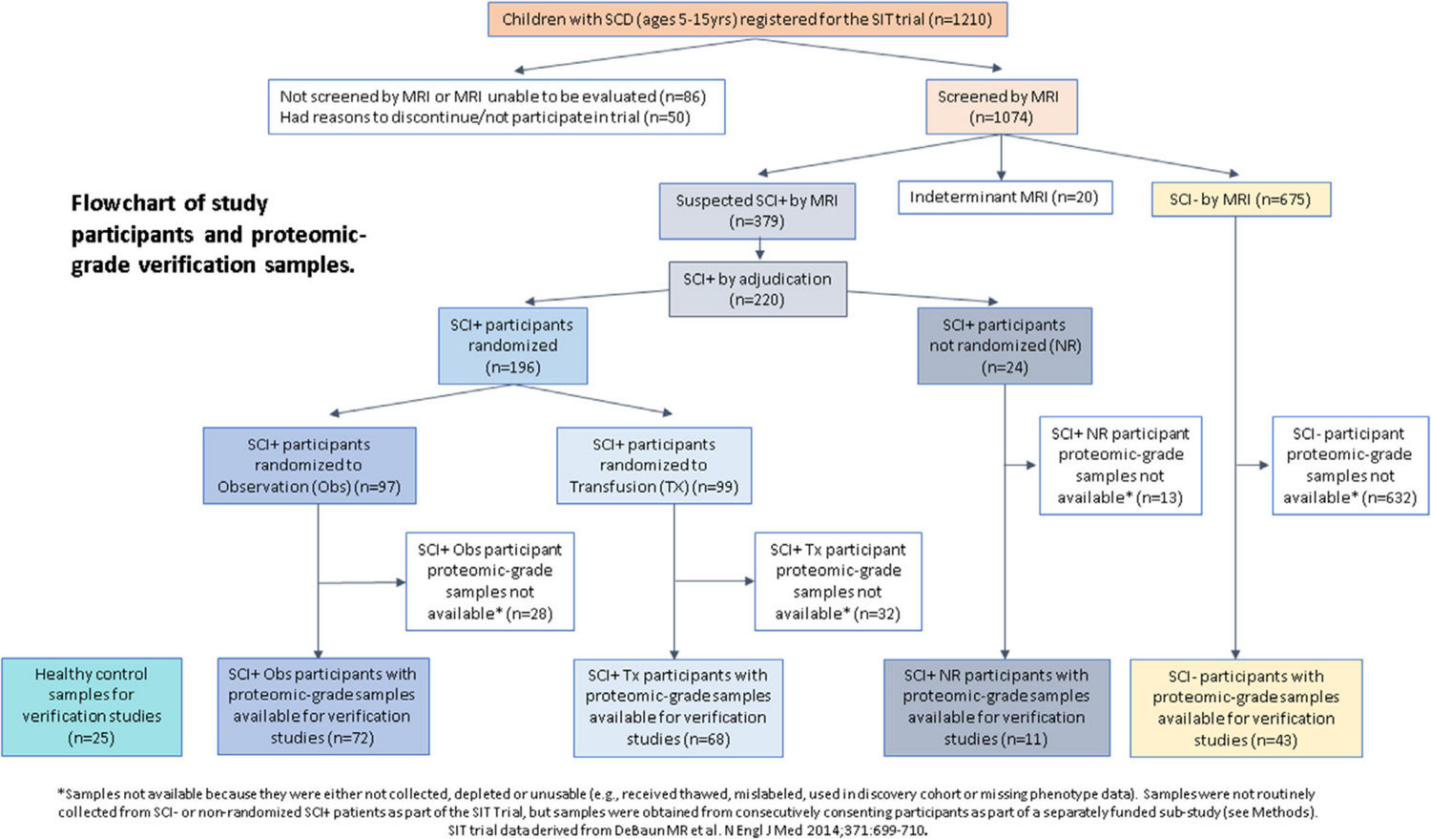
Flow chart of participants samples selected for verification analysis

For verification of the MS-identified protein NRGN, stored plasma samples obtained at various timepoints during the SIT Trial were tested for NRGN levels by immunoassay. The majority of these samples were from an ancillary study to collect longitudinal samples from SIT Trial participants that started after the SIT Trial had begun, at a subset of sites that agreed to participate. Groups of subjects in the treatment (*n* = 68), observation (*n* = 72), declined randomization (*n* = 11), and SCI negative (*n* = 43) groups had high quality proteomic samples collected longitudinally at 0, 6, 12, 24, and 36 months after enrollment. Eighty-nine participants (43 observation, 46 treatment) exiting during the ancillary study had proteomic grade exit samples; 30 participants had standard entry plasma samples that were analyzed. There was no overlap between the discovery group and the verification group samples. Samples that were not collected or handled according to protocol were not included in this study; all other samples were included. An additional 25 cross-sectional plasma samples from healthy, age, gender, and race comparable non-SCD pediatric controls unrelated to the SIT Trial, without evidence of acute/chronic illness (except for asthma, attentiondeficit-hyperactivity disorder (ADHD), mood disorders, bipolar disorder, sleep disorders, allergies, iron deficiency, thyroglossal duct cyst, and esotropia) were obtained from the Harriet Lane Pediatrics Clinic at the Johns Hopkins Hospital through a separate IRB-approved study. Figure 1 shows a flowchart of the study participants.

### 2.2 | Sample preparation and hemoglobin depletion

SIT Trial screening samples collected at study entry were shipped and stored at room temperature (storage time median 2 days, range 1 to 6 days), aliquoted and frozen at −80°C. Longitudinal proteomic-grade samples and healthy control participant samples were frozen at 80°C on site within a 4 h window after phlebotomy, and processed per the SIT Trial protocol [18]. Obvious hemolysis was observed in the SIT screening discovery SCD samples. To enrich for low abundance proteins, Hb was depleted from SCD plasma samples using nickel-nitrilotriacetic acid (Ni-NTA) beads (Qiagen) [19]. Non-SCD discovery plasma samples had no observable hemolysis and were not subjected to this depletion step. In a separate study, NRGN levels were found to be stable after sitting at room temperature for 4 days [20].

### 2.3 | Plasma abundant protein depletion and fractionation

Using the ProteomeLab IgY-12 LC10 column kit (Beckman Coulter, Inc., Fullerton, CA) and the manufacturer’s protocol, samples underwent immunoaffinity depletion of the top 14 abundant plasma proteins [21]. Subsequently, 400 *μ*g of the depleted protein samples were separated by reversed phase HPLC using PS-HPRP 2D (4.6 × 33 mm) columns (Beckman-Coulter, Inc.), also on a PF 2D LC platform (Beckman Coulter, Inc., Fullerton, CA). Solvent A was composed of 0.1% TFA in water and solvent B was 0.08% TFA in acetonitrile. The AB gradient was run from 5 to 15% B in 1 min, 15 to 25% in 2 min, 25 to 31% in 2 min, 31 to 41% in 10 min, 41 to 47% in 6 min, 47 to 67% in 4 min, finally up to 100% B in 3 min, held for 1 min, and back to 5% in 1 min at a flow rate of 1 mL/min. The resulting 39 reversed phase (RP)-HPLC fractions were collected in 1 mL 96-well plates. The fractionated proteins were neutralized, vacuum-dried, digested with sequencing-grade modified trypsin (Promega, Madison, WI) and desalted according to Sheng et al. [21].

### 2.4 | MS analysis for protein identification

Tandem (LC-MS/MS) experiments were performed on a linear trap quadrupole (LTQ)-Orbitrap ELITE mass spectrometer (ThermoFisher, San Jose, CA) equipped with an on-line nano-HPLC (Agilent Technologies, 1200 Series, Wilmington, DE), as previously described [19]. The MS raw data were analyzed using Proteomics Alternative Splicing Screening (PASS) (Integrated Analysis, Bethesda, MD) with X!Tandem searches (www.thegpm.org; version 2008.12.01) of the non-redundant International Protein Index (IPI) peptide database (human, 3.19). Peptide identifications were accepted if they could be established at greater than 95% probability and contained at least 2 unique identified spectra per peptide [22], with probability based Mowse scores greater than 35 (*p* < 0.05) and charge of >+2. To remove protein name redundancy, the dataset was filtered based on 90% amino acid sequence homology using cluster database at high identity with tolerance (CD-HIT) [23]. All single peptide proteins had their MS spectrum manually validated. All isoforms were identified based on observed peptide to an amino acid sequence that is unique to the specific isoform.

### 2.5 | Brain-enriched protein database

To develop a brain-enriched protein list to query our plasma MS dataset, publicly available data sources for oligonucleotide microarray (http://www.genecards.org/index.shtml), expressed sequence tags (EST) (https://ncbiinsights.ncbi.nlm.nih.gov/2019/07/30/the-unigene-web-pages-are-now-retired) and serial analysis of gene expression (SAGE) databases (https://mitelmandatabase.isb-cgc.org) were used to identify proteins that are specific or enriched in the brain. When data were available, the Human Protein Atlas (http://www.proteinatlas.org/) was also used to confirm enriched brain protein expression.

Proteins were given a brain-enriched score based on their relative expression of transcripts in human brain, and 27 other normal human tissues as assessed by available microarray (www.biogps.com), EST (National Center for Biotechnology Information - NCBI), and SAGE (NCBI) data. Scoring criteria included: microarray data showing greater than ten-fold increase in expression over baseline, EST and SAGE data showing presence of the protein in less than two other tissues. Proteins received either a score of 1 or 0 for each category, with a maximum score of 3 when all three brain enrichment categories were met. A composite list of brain proteins meeting these criteria was used to filter the MS data to identify brain proteins in children with and without SCD and SCI.

### 2.6 | Ingenuity pathway analysis

Ingenuity Pathway Analysis (IPA) program (http://www.ingenuity.com) was used to analyze the pathway network of the proteins with abundance changes that were identified through MS. The protein accession numbers and corresponding expression values were uploaded as an Excel spreadsheet file into the Ingenuity software, which algorithmically generate networks between proteins with differential expression using the Ingenuity Knowledge Base. Each network is assigned a score, used to rank networks according to their relevance to the proteins in the dataset. A score > 2 is considered as a valid network. Identified networks were analyzed to rank significant biological functions. Biological functions were categorized into diseases/disorders, molecular/cellular functions, and physiological system development/function. Canonical pathways were grouped in metabolic and signaling pathways. Right-tailed Fisher’s exact tests were used to calculate p values to determine the probability of network assignment due to chance.

### 2.7 | Neurogranin (NRGN) ELISA

A human NRGN ELISA that our group developed was used as previously described [24], an electro-chemiluminescent sandwich immunoassay for NRGN based on the MesoScale Discovery platform (MesoScale Discovery, Gaithersburg, MD). A purified, mouse monoclonal anti-NRGN was used as the capture antibody, and an unlabeled polyclonal rabbit anti-NRGN was used for detection, and identified by a MesoScale Discovery Sulfo-TAG-labelled goat anti-rabbit antibody (R32AB). The standard curve (from 40–0.055 ng/mL) was constructed by serial dilutions of purified recombinant hNRGN in 1 X PBS containing 1% bovine serum albumin (SeraCare Life Sciences, Milford, MA).

### 2.8 | Statistical analyses

For the verification group, NRGN immunoassay concentration levels were analyzed in duplicate using parametric and non-parametric statistical tests to compare groups. We compared longitudinal changes in plasma concentration differences for NRGN using a multi-level mixed effects linear regression model. Spearman test was used to analyze the correlations between NRGN concentrations and other variables. Sample assays were repeated with appropriate controls if values had a coefficient of variation (CV%) greater than 20%. The average lower limit of quantification for the assay was 0.039 ng/mL and the average lower limit of detection for the assay was 0.012 ng/mL. Values of NRGN that were below the lower limit of quantification of the assay, but above the lower limit of detection of the assay, were recorded as half of the value of the lower limit of quantification for the assay. We also did a sensitivity analysis to look at the impact of processing time duration on NRGN values. All samples processed over a period of 4 days or greater were withheld while statistical analyses were repeated. A p value less than 0.05 was considered statistically significant. Statistical analyses were conducted using Stata version 11.0 (StataCorp., College Station, Texas).

## 3 | RESULTS

### 3.1 | Baseline characteristics of children with SCD and controls

Characteristics and differences between the discovery groups are presented in Tables 1 and 2. The SCI positive group had a total of 28 hospitalizations/emergency department visits for SCD related issues (pain crises, acute chest syndrome, asthma/respiratory symptoms) vs. 40 visits for the SCI negative group. The SCI positive group had a total of 57 lesions (mean 8.1, range 2 to 14), with an average total lesion volume of 7.4 (range 5.2 to 10.8). Data on lesion size was not available for one participant. None of the participants were on hydroxyurea at the time of screening for the SIT Trial.

### 3.2 | Characterization of the plasma proteome of children with SCD

**FIGURE 2:**
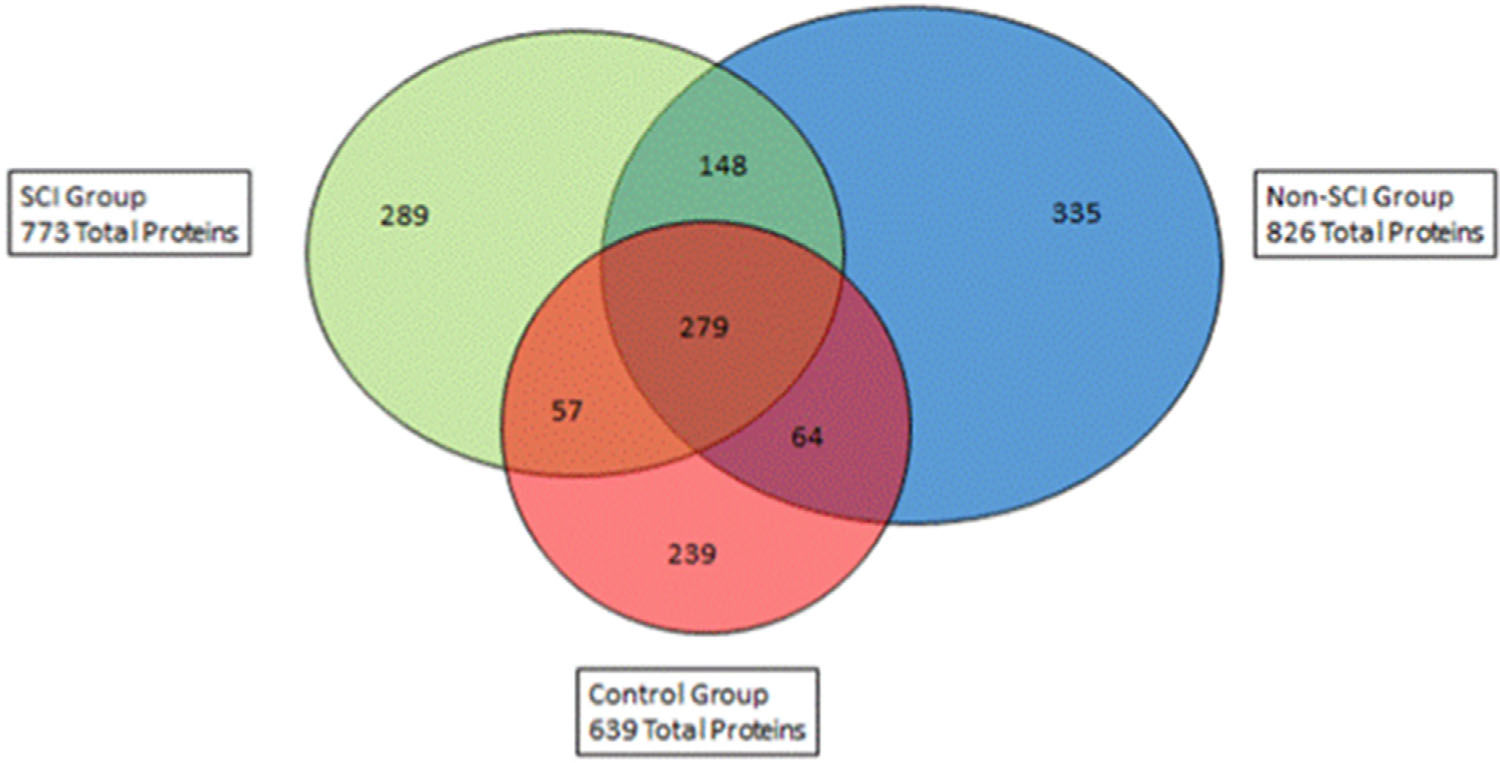
Venn diagram of the number of proteins identified and the overlap in the normal, non-silent cerebral infarction and silent cerebral infarction groups. SCI – Silent Cerebral Infarction.

In all, 819 fractions were quantified using LC-MS yielding 672460 spectra. Using X! Tandem searches of the IPI Proteomics and Uniprot databases, we identified a total of 1172 unambiguous proteins in the plasma proteome of children with SCD (Figure 2 and Table S1). Excluding the proteins found in the control group, the SCI group uniquely contained 25% (289/1172), the SCI negative group uniquely contained 29% (335/1172) of these proteins, and 13% (148/1172) of proteins were common to both groups. Of the proteins identified, 239 proteins were found only in healthy controls, and not in SCD participants (Figure 2 and Table S2).

There were 23 proteins detected in at least two individuals of the SCI positive and SCI negative groups with spectral counts greater than two-fold difference between the two groups (Table 3). Inflammatory pathway proteins were commonly elevated in the SCI negative group, including L-selectin, a homing receptor for leukocytes to endothelial cells [25] and S100A11, a ligand for the receptor for advanced glycation end products (RAGE) receptor [26]. Complement proteins were also increased in the SCI negative group, including complement proteins C1q subcomponent subunits A and B [27], C4b-binding protein beta chain [28] and C8 gamma chain [29]. Platelet basic protein (CXCL7), a platelet-derived chemokine that functions to activate and attract neutrophils [30], was abundantly elevated in the SCI negative group. Elevated levels of teneurin-3 (TEMN3), involved in connectivity and axon guidance [31], were seen in the SCI negative group (6.5 fold) and cell death regulator Aven (AVEN), an apoptosis and caspase activation inhibitor [31], in the SCI positive group (2.5 fold).

Analysis using IPA revealed that the proteins identified in SCD plasma demonstrated overrepresentation of a number of biological pathways. Neurological disease was ranked among the top 5 diseases in the SCI positive group, but not in the SCI negative group. Additional IPA analysis revealed that proteins identified in the SCI group pathways are involved in more specific disease processes that have already been implicated in SCD, namely ischemia-reperfusion injury [32,33], endothelial dysfunction [34] and neuronal injury and death [35]. Specific protein pathways linked by IPA in this study include: (1) tauopathy (microtubule-associated protein tau [MAPT] and glial fibrillary acidic protein [GFAP]), (2) axon loss (MAPT) and (3) cerebral amyloid angiopathy (cystatin-C [CST3] and vimentin [VIM]).

### 3.3 | Identification of brain proteins

An iterative process was used to identify circulating brain proteins from our discovery cohort. A review of publicly available oligonucleotide microarray, EST and SAGE databases identified 524 genes with increased messenger ribonucleic acid (mRNA) expression in brain (Table S3). Our MS protein identification data were filtered against this list of expressed brain proteins to produce a composite list of brain proteins found in plasma from children with SCD, but not found in plasma from age, gender and race-matched healthy control children. Using this methodology, we identified a total of 25 brain-specific proteins in plasma from children with SCD (Table 4). When we filtered the MS protein identification data for age-matched non-SCD controls against the list of expressed brain proteins listed in Table S3, we identified two brain-specific proteins: low density lipoprotein receptor-related protein 4 (LRP4; accession # O75096) and rabphilin (RPH3A accession# Q9Y2J0). These proteins were not found in plasma from children with SCD.

These brain-specific proteins are derived from both neuronal and astrocyte/glial cells across the brain. The proteins identified encompassed all cell compartments, but were predominately membrane-bound (11/25, 44%) and identified at relatively low levels (1 peptide, 18/25, 72%), as would be expected for a brain protein in plasma. The significance of low level detection in SCD has been demonstrated for GFAP, which was identified at the 2 peptide level [18,35]. The most abundant (spectral count = 15) brain protein identified in the plasma of children with SCD was NRGN, a small (7.6 kilodalton, 78 amino acids) calcium-dependent neuronal signaling protein not previously identified in plasma from patients with SCD [36]. Therefore, we developed an ELISA for NRGN for verification as described in Yang et. al. [24].

### 3.4 | Plasma NRGN levels in the verification cohort

We used the NRGN ELISA to verify the discovery proteomic data with plasma samples from the SIT Trial Biologic Repository from children with SCD and SCI (*n* = 152) and no SCI (*n* = 43), as well as from healthy children (*n* = 25). Table 5 shows the characteristics of the entire group of participants.

**FIGURE 3:**
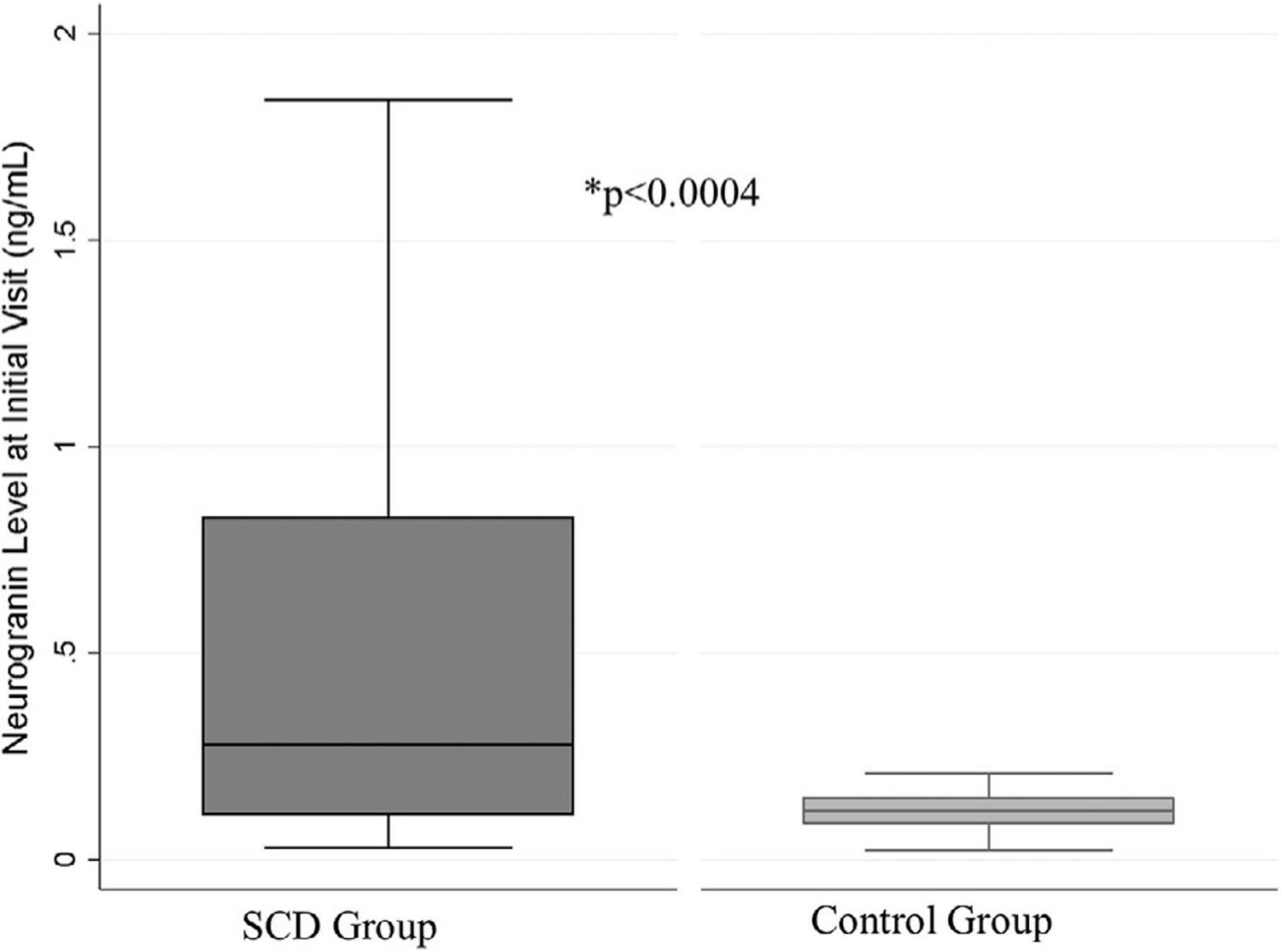
Median neurogranin levels by sickle cell disease status. Box plot of neurogranin levels from a group of children with sickle cell disease (*n* = 104) and a group of healthy pediatric control participants (*n* = 25). Line in middle of box represents median neurogranin level. Horizontal line at top of box represents 75^th^ percentile value. Horizontal line at bottom of box represents 25^th^ percentile value. Line at top of whisker represents upper adjacent value. Line at bottom of whisker represents lower adjacent value. Outliers were removed from the graph. SCD – Sickle Cell Disease. NRGN – Neurogranin.

Using initial study visit samples (earliest available sample from either the participant’s screening or baseline visit), there was a significant difference in median NRGN levels between the SCD (*n* = 101) and pediatric healthy control groups (*n* = 25), (0.28 vs. 0.12 ng/mL, 25–75%IQR: 0.11–0.83 vs. 0.09–0.15 ng/mL, *p* < 0.0004) (Figure 3). Using the initial study visit samples from the SIT Trial, there was no significant difference in median NRGN levels between the SCI negative (*n* = 34) and SCI positive groups (*n* = 67) (0.54 vs 0.2 ng/mL, 25–75%IQR: 0.1–1.01 vs. 0.11–0.66 ng/mL, *p* = 0.27, 0.21 in sensitivity analysis). Given the expected significant difference in age between the SCI positive and SCI negative groups (Table 5), and as age is a known risk factor for SCI, we compared the mean age at the initial visit between the SCD and pediatric healthy control group and did not find a significant difference between the groups (111.4 vs. 126.5 months, 95% CI: 104.2–118.7 vs. 110.9 – 142.1, *p* = 0.07).

As shown in the supplemental analyses, there was no association of NRGN with age, neuropsychological measures of executive function or change over time in SCI or non-SCI groups.

## 4 | DISCUSSION

Biomarkers of subclinical brain injury in SCD are needed to diagnose and monitor therapy and disease progression, as well as aid in the development of molecular targeted therapies. Proteomics provides an opportunity to discover these biochemical markers in complex mixtures, such as plasma. Proteomic techniques have been used for biomarker discovery of brain proteins in a number of disease states, including brain cancer [37,38], Alzheimer’s disease [39,40], traumatic brain injury (TBI) [41,42], and stroke. [43,44]. However, very few studies have used plasma proteomics for clinical biomarker discovery in SCD [45]. We used a proteomic-based approach to test the hypothesis that children with SCD with and without SCI have brain proteins circulating in their plasma proteome that are associated with subclinical brain injury. We also explored the hypothesis that difference would be seen between children with SCD and normal control participants. We then verified our experimental identification of one circulating brain protein, NRGN, using longitudinal samples from children with SCD and SCI, children with SCD and without SCI, and healthy control participants.

Limited information is available regarding circulating biomarkers for neurologic and other complications of SCD. Using a targeted candidate approach, we have previously reported associations between the vascular stress proteins thrombospondin and L-selectin with SCI in SCD as well as neuronally secreted brain-derived neurotrophic factor (BDNF) in SCD participants in comparison to control participants [46,47]. As described in this study, we pursued a non-biased approach to identify circulating proteins that could differentiate SCD patients at risk of SCI. There were 239 unique proteins identified in the SCI discovery group. Similarly, Tewari et al. found elevated levels of 13 proteins in SCD pediatric participants with SCI in comparison to SCI negative participants with SCD, including one protein, fibrinogen gamma chain, which we also found to be elevated in our SCD group per spectral counts [48]. Kakhniashvili et al. used two-dimensional fluorescence difference gel electrophoresis (2D DIGE) and tandem MS (LC-MS/MS) to evaluate quantitative changes in the red blood cell (RBC) membrane proteome and described elevations of proteins involved in repair in SCD after oxidative stress [49]. Others have used SELDI-TOF and MALDI-TOF MS to evaluate biomarkers of pulmonary hypertension [50] and acute painful episodes [51] in SCD.

In our proteomics study, use of complementary and overlapping mRNA/protein databases (SAGE, EST, microarray and Human Protein Atlas) identified 524 expressed genes enriched in the brain. This brain-enriched gene list may implicate neuroaxonal injury in the pathophysiology of subclinical brain injury in children with SCD. Potential mechanisms for this neuroaxonal damage include mitochondrial injury and defects in calcium homeostasis [52] (copine-6), damaging changes in sodium channel function [53] (amiloride-sensitive cation channel 4), glutamate receptor activation [54] (glutamate receptor, metabotropic 4 variant), invading proteolytic enzymes [55] (*β*-Ala-His dipeptidase/carnosinase), neuronal damage (NRGN) [56], as well as impaired neurite outgrowth [57] (SLIT and Neurotrophic Tyrosine Kinase Receptor [NTRK]-like protein 3), neuronal differentiation [58,59] (transcription factor SRY-Box Transcription Factor 3 [SOX]−3 and neuronal membrane glycoprotein M6-a).

The results from IPA suggest that children with SCD are at risk for neuronal injury and cell death, through tauopathy and axonal loss. These analyses suggest that the presence in plasma of GFAP, a known biomarker of stroke and traumatic brain injury, could be due to brain injury in children with SCD and SCI. GFAP is elevated in participants with SCD when compared to healthy controls and associated with ischemic brain injury, and inversely correlated with performance IQ [18,35]. Similarly, MAPT, an axonal cytoskeletal protein that has been implicated in several neurodegenerative disorders [60] and TBI [61], was detected in the plasma of SCD children (average spectral count = 2). Abnormal phosphorylation of MAPT can lead to the formation of neurotoxic insoluble tau aggregates, which results in loss of neurons [62]. The identification of MAPT suggests a potential role for axonal loss in the pathophysiology of SCI in children with SCD. Furthermore, CST3, a basic protein that inhibits cysteine proteases implicated in cerebral amyloid angiopathy and neuroprotective in TBI [63,64], was identified in both SCI positive and SCI negative groups (average SC = 7.2). CST3 has been used as an indicator of renal glomerular dysfunction in participants with SCD [65,66], but has not been studied in subclinical brain injury in SCD.

We measured our most abundant MS discovery protein, NRGN, with a new ELISA in a cohort of children with SCD from the SIT Trial and a group non-SCD control children of similar age, gender and race. NRGN levels were significantly different between non-SCD controls and SCD participants at enrollment. When studied longitudinally in SIT participants, NRGN levels were not significantly different between the SCI observation and SCI transfusion treatment groups and did not significantly change over time.

The significance of elevated levels of NRGN, a neuron-specific signaling protein, in the blood of children with SCD is presently unknown; however, circulating levels of NRGN likely reflect cellular injury, especially necrosis. Elevated levels of NRGN have been found in the serum of individuals with TBI [24] and plasma NRGN levels correlate with infarct volume in adult acute ischemic stroke patients [67]. Most studies have investigated NRGN genetic polymorphisms in adults with schizophrenia [68–70] and in cerebrospinal fluid (CSF) in Alzheimer’s disease [71,72], relating NRGN to learning and memory impairment [73–75]. Another study noted that NRGN levels decreased in cognitively intact older adults using two samples collected between 3 and 11 year intervals [76]. NRGN is of particular interest in regards to SCD, as a calcium-sensitive, calmodulin-binding,neuron-specific signaling protein, which has been implicated in synaptic development and remodeling [73], thyroid hormone signaling [77], stroke [67] and learning [78]. Its role in cognition is also demonstrated in NRGN knockout mice, which have structurally normal brains, but considerable learning deficits [79]. While we did not see differences in NRGN levels between participants with SCD with and without SCI, this could be due to the timing of the blood draws or the sensitivity of the NRGN assay may not be able to discriminate the SCI− and SCI+ groups.

The conclusions of this evaluation are limited by several factors. For example, the SIT Trial samples were not designed to measure time-dependent correlations with acute brain injury. Also, children with the highest risk of stroke (elevated transcranial Dopper [TCD] velocity) were excluded from the trial, which precludes us from determining a causal relationship between TCD velocities and NRGN or other lead proteins identified. In addition, a small number of samples were used for the initial proteomic discovery analysis and a limited number of control samples were available for the verification assays, though matched for group characteristics. The amount of mass spectrometry time required for the study design precluded doing larger sample sizes, however, the initial step is intended only for identification of candidate proteins, and the study design compensates for weaknesses of small sample size during discovery with larger subsequent validation cohorts. Furthermore, we have previously shown that the use of nickel beads for Hb depletion makes relative concentration determinations of some proteins challenging [19]. Hb depletion using Ni-NTA beads was only done in the SCD group, as excess plasma Hb was not present in the control group; therefore, our list of brain proteins in plasma from children with SCD may not be exhaustive for proteins involved in the pathophysiology of subclinical brain injury, and ratios of spectral counts after depletion may have been affected by plasma hemoglobin levels. This may have contributed to discrepant results between the discovery cohort and the verification results and the L-selectin levels between the current study and our prior results (L-selectin levels were higher in the plasma of individuals with SCI compared to those with no SCI in the prior study [47], whereas spectral counts were lower in patients within SCI in the current study). These differences may also reflect the limitation of quantitation of protein levels using spectral counts; however, these factors should not have affected the results of the verification assays in the current study [19].

In summary, we have developed and verified a proteomic workflow for brain biomarker discovery in children with SCD. We are the first to report significant elevations of NRGN in children with SCD as compared to non-SCD controls. While this study focused largely on NGRN, the ultimate value of this study may be in the numerous other brain proteins potentially involved in brain injury in SCD that deserve additional investigation. Collectively, these findings support further proteomic discovery research in children with SCD, which may provide new biomarkers for determining extent of disease, following the course of injury and response to therapy, predicting brain injury and establishing potential targets for therapeutic drug discovery.

## Supplementary Material

Supplemental Data

Supplemental Table 1

Supplemental Table 3

Supplemental Table 2

## Figures and Tables

**TABLE 1:** Clinical characteristics of children with sickle cell disease and healthy, non-sickle cell disease control participants, used for proteomic discovery analysis

Clinical characteristics	SCD [*n*= 15]	Control group [*n*= 6]
% Sickle cell trait [*n*]*	0 [15]	50 [3]
% Male [*n*]	67 [10]	50 [3]
Mean age (SD)	9.4 (2.7)	11.5 (2.1)
Median reticulocyte % (IQR)	10.3 (8.2–12.2)	7.5 (5.5–12)
Median hemoglobin g/dL (IQR) *	8.9 (7.6–9.1)	12.05 (11.8–12.4)
Median hematocrit % (IQR) *	24 (22–25)	36.45 (34.8–37.8)
Median WBC x10^9^/L (IQR) *	12.8 (10–17.3)	4.9 (4.3–5.5)
Median platelet x10^9^/L (IQR) *	445 (368–570)	327.5 (319–332)

Abbreviations: SCD, sickle cell disease; SD, standard deviation; IQR, interquartile range; WBC, white blood cell count.

Results represent mean ± standard deviation (SD).

* p < 0.05 between groups by Student’s *t*-test.

**TABLE 2:** Clinical characteristics of children with SCD with and without SCI, used for proteomic analysis

Clinical characteristics	SCI positive [*n*= 7]	SCI negative [*n*= 8]
% Male [*n*]	71 [5]	63 [5]
Mean age (SD)	9.8 (2.4)	9 (3.1)
Median reticulocyte % (IQR)	10.7 (9.4–13.1)	9.6 (7–12)
Median hemoglobin g/dL (IQR)	8.4 (7.4–9.1)	8.9 (8.2–9)
Median hematocrit % (IQR)	24 (21–25)	24.5 (22.5–25)
WBC x10^9^/L (IQR)	13 (9.27–23.4)	12.8 (11–14)
Platelet x10^9^/L (IQR)	418 (335–456)	516 (437–597)

Abbreviations: IQR, interquartile range; SCI, silent cerebral infarction; SD, standard deviation; WBC, white blood cell count.

Results represent median (25% Interquartile range [IQR] – 75% IQR) unless otherwise specified.

* p < 0.05 between groups by Student’s *t* test.

**TABLE 3:** Protein differences (>2 < 0.5-fold spectral counts) identified in SCI positive and SCI negative SCD groups

Accession #	Protein name	Total samples (SCI negative)	Total samples (SCI positive)	Avg SC/protein (SCI negative)	Avg SC/protein (SCI positive)	SC Ratio (SCI negative/ SCI positive)
Q9P273	Teneurin-3	7.0	3.0	51.9	8.0	6.5
P31949	Protein S100-A11	4.0	2.0	41.5	7.5	5.5
P16401	Histone H1.5	2.0	2.0	21.0	4.5	4.7
Q9UJ43	L-selectin	8.0	2.0	17.8	4.0	4.4
P50552	Vasodilator-stimulated phosphoprotein	5.0	3.0	15.8	4.0	4.0
P18206	Vinculin	7.0	3.0	34.3	10.3	3.3
P10599	Thioredoxin	7.0	2.0	44.1	13.5	3.3
P20851	C4b-binding protein beta chain	8.0	5.0	22.1	7.8	2.8
P02775	Platelet basic protein	8.0	7.0	174.7	65.0	2.7
P02745	Complement C1q subcomponent subunit A	5.0	2.0	19.4	7.5	2.6
Q9UK55	Protein Z-dependent protease inhibitor	6.0	4.0	19.5	8.3	2.4
Q92954	Proteoglycan 4	8.0	6.0	26.2	11.7	2.2
P02746	Complement C1q subcomponent subunit B	8.0	7.0	105.4	47.0	2.2
Q96IY4	Carboxypeptidase B2	7.0	4.0	14.3	6.5	2.2
P07360	Complement component C8 gamma chain	8.0	7.0	53.2	25.3	2.1
P02533	Keratin, type I cytoskeletal 14	7.0	5.0	43.6	20.8	2.1
P08670	Vimentin	5.0	4.0	12.6	6.3	2.0
Q96PD5	N-acetylmuramoyl-L-alanine amidase	8.0	7.0	305.0	153.0	2.0
Q6B823	Histone H4	3.0	2.0	5.0	10.0	0.5
Q96F45	Zinc finger protein 503	3.0	2.0	2.0	4.0	0.5
P02679	Fibrinogen gamma chain	8.0	7.0	92.1	185.9	0.5
Q9NQS1	Cell death regulator Aven	3.0	2.0	4.0	10.0	0.4
P02461	Collagen alpha-1(III) chain	3.0	2.0	3.7	9.5	0.4

Abbreviations: SCI, silent cerebral infarction; SCD, sickle cell disease; SC, spectral counts; Samples, total number of samples where protein is identified.

**TABLE 4:** Top 25 brain proteins identified in children with SCD

Accession #	Protein name	Protein function	Cellular component	Average SC per Protein	Log(e)
**Q92686**	Neurogranin	Acts as a “third messenger” substrate of protein kinase C-mediated molecular cascades during synaptic development and remodeling. Binds to calmodulin in the absence of calcium	Membrane; Cytoplasm	15	−6.40
**Q96KN2**	Beta-Ala-His dipeptidase	Preferential hydrolysis of the beta-Ala-|-His dipeptide (carnosine)	Secreted	7.25	−25.08
**Q99884**	Sodium-dependent proline transporter	Terminates the action of proline by its high affinity sodium-dependent reuptake into presynaptic terminals.	Membrane	6	−2
**P01213**	Beta-neoendorphin-dynorphin	Pain perception and responses to stress	Secreted	2	−9.9
**P56975**	Pro-neuregulin-3, membrane-bound isoform	Direct ligand for the ERBB4 tyrosine kinase receptor	Membrane	2	−1.10
**P14136**	Glial fibrillary acidic protein	Distinguishes astrocytes from other glial cells during the development of the CNS	Cytoplasm	2	−6.33
**O94933**	SLIT and NTRK-like protein 3	Suppresses neurite outgrowth	Membrane	2	−5.3
**P08908**	5-hydroxytryptamine receptor 1A	Serotonin receptor	Membrane	1	−1.1
**A7E2E4**	Dipeptidyl-peptidase 6	May modulate the cell surface expression and the activity of the potassium channel KCND2	Membrane	1	−1.1
**Q96FT7**	Amiloride-sensitive cation channel 4	Cation channel with high affinity for sodium	Membrane	1	−1.2
**B3KXG7**	Protein tyrosine phosphatase, non-receptor type 5	Hydrolase receptor	Endoplasmic reticulum membrane	1	−1.5
**Q9P218**	Collagen alpha-1(XX)	Collagen protein	Secreted	1	−1.5
**O95741**	Copine-6	Membrane trafficking and in synaptic plasticity	Mitochondrion	1	−1.2
**Q9UI47**	Catenin alpha-3	Formation of stretch-resistant cell-cell adhesion complexes	Cytoplasm	1	−1.3
**P51674**	Neuronal membrane glycoprotein M6-a	Neuronal differentiation, including differentiation and migration of neuronal stem cells	Membrane	1	−1.4
**Q9UQM7**	Calcium/calmodulin-dependent protein kinase type II subunit alpha	Long-term potentiation and neurotransmitter release	Membrane	1	−1.2
**Q96NJ5**	Kelch-like protein 32	Unknown	Unknown	1	−2
**Q8N967; Q6ZUR1**	Leucine-rich repeat and transmembrane domain-containing protein 2	Unknown	Membrane	1	−1.4
**Q96NK8**	Neurogenic differentiation factor 6	Trans-acting factor involved in the development and maintenance of the mammalian nervous system	Nucleus	1	−1.5
**Q8N987**	N-terminal EF-hand calcium-binding protein 1	Calcium ion binding	Cytoplasm	1	−1.6
**Q13516**	Oligodendrocyte transcription factor 2	Oligodendrocyte and motor neuron specification in the spinal cord; adevelopment of somatic motor neurons in the hindbrain	Nucleus; Cytoplasm	1	−1.1
**Q59GK5**	Glutamate receptor, metabotropic 4 variant	Receptor	Membrane	1	−1.4
**Q16650**	T-box brain protein 1	Transcriptional regulator of brain development	Nucleus	1	−1.8
**Q504Y0**	Zinc transporter ZIP12	Zinc-influx transporter	Membrane	1	−1
**P41225**	Transcription factor SOX-3	Required for formation of hypothalamo-pituitary axis; counteracts the activity of proneural proteins and suppresses neuronal differentiation	Nucleus	1	−1.30

Abbreviations: SCD, sickle cell disease; SC, spectral counts; CNS, central nervous system; Log(e), natural logarithm

**TABLE 5:** Clinical characteristics of children with SCD and healthy, non-SCD controls, from verification analysis

	All SCD subjects	Treatment group	Observation group	SCI positive	SCI negative	Control group
**Total *n***	194	68	72	151	43	25
**SCD type**						
HbSS	175	62	64	136	39	Unknown
HbS-*β* thal	11	3	4	8	3	
**Male** (%)	107 (55)	41 (60)	39 (54)	88 (58)	19 (44)	9 (36)
**Mean BMI** in kg/m^2^ (SD)	16.86 (2.76)	16.72 (2.17)	17.15 (3.22)	16.9 (2.71)	16.65 (3.14)	
**Mean age at initial study visit in months** (SD)	111.4 (36.8)	117.4 (34)	121.1 (41.1)	118.9 (37.4)^a^	96.7 (31.3)^a^	126.5 (37.9)
**Race** (%)						
Black	177 (91)	62 (91)	66 (92)	139 (92)^b^	38 (88)^b^	25 (100)
Asian	1 (1)	6 (9)	1 (1)	1 (1)	1 (2)	
Pacific Islander	1 (1)		5 (7)	11 (7)	4 (9)	
White						
Other	15 (8)					
**Ethnicity**						Unknown
Hispanic	3	1	1	2	1	
Not Hispanic	189	67	71	148	41	
Unknown	2			1	1	
**History of** (%)						
**Asthma**	47 (24)	17 (25)	20 (28)	38 (25)	9 (21)	10 (40)
**ADHD**						4 (16)
**Sleep disorder**						2 (8)
**TCD status** (%)						
Normal	144 (74)	52 (76.5)	57 (79)	118 (78)	26 (60)	Not applicable
Conditional	26 (13)	13 (19)	11 (15.5)	24 (16)	2 (5)	
High	5 (3)	2 (3)	1 (1.5)	4 (3)	1 (2)	
Missing	19 (10)	1 (1.5)	3 (4)	5 (3)	14 (33)	
**Median steady state hemoglobin** in g/dL	7.8	7.8	7.9	7.8	7.7	Unknown
(25%−75%IQR)	(7.4–8.7)	(7.2–8.4)	(7.5–9.1)	(7.4–8.8)	(7.5–8.7)	
**Median % Steady State Reticulocytes**	11.2	12.5^2^	10^2^	11.2	11.6	Unknown
(25%−75%IQR)	(8.6–16.2)	(9.6–16.8)	(7.6–13.6)	(8.2–16.3)	(10.6–14.6)	
**Median platelet count** per mm^3^	435000	413000	446500	433000	475000	Unknown
(25%−75%IQR)	(380000–526000)	(376000–513000)	(371500–520000)	(376000–520000)	(391500–578500)	
**Median % Hgb F**	7.2	6.3	6.5	6.5	8.9	Not Applicable
(25%−75%IQR)	(4.2–12.2)	(4–11.7)	(4.8–11.8)	(4–12.2)	(5–14.4)	
	(*n* = 96)	(*n* = 38)	(*n* = 37)	(*n* = 80)	(*n* = 16)	
**Median steady state WBC** per mm^3^	12445	12250	12100	12445	12450	Unknown
(25%−75%IQR)	(9950–14400)	(9265–14400)	(9550–14180)	(9550–14400)	(10550–14550)	

Abbreviations: SCD, sickle cell disease; SD, standard deviation; WBC, white blood cell count; TCD, transcranial Doppler

a *p* < 0.004.

b *p* < 0.02.

## Data Availability

The SIT Trial data used in this manuscript are available on request from the corresponding author. The MS data are not publicly available due to patient consent issues.

## Statement of Clinical Relevance

Limited proteomic discovery work has been done involving sickle cell disease and neurological complications. This study identified multiple new proteins of interest with regards to silent cerebral infarction in pediatric sickle cell disease. An ELISA assay was used to measure the levels of one protein, neurogranin, a neuronal protein. Neurogranin levels were elevated in children with sickle cell disease in comparison to healthy control participants.

## Abbreviations:

SCD: sickle cell disease
SCI: silent cerebral infarcts
MRI: magnetic resonance imaging
NRGN: neurogranin
SIT Trial: Silent Cerebral Infarct Multi-Center Clinical Trial
Hb: haemoglobin
WBC: white blood cell counts
ADHD: attention-deficit-hyperactivity disorder
Ni-NTA: nickel-nitrilotriacetic acid
RP: reversed phase
LTQ: linear trap quadrupole
PASS: Proteomics Alternative Splicing Screening
IPI: international protein index
CD-HIT: cluster database at high identity with tolerance
EST: expressed sequence tags
SAGE: serial analysis of gene expression
NCBI: National Center for Biotechnology Information
IPA: Ingenuity Pathway Analysis
CV: coefficient of variation
RAGE: receptor for advanced glycation end products
CXCL7: platelet basic protein
TEMN3: teneurin-3
AVEN: aven
MAPT: microtubule-associated protein tau
GFAP: glial fibrillary acidic protein
CST3: cystatin-C
VIM: vimentin
mRNA: Messenger ribonucleic acid
LRP4: low density lipoprotein receptor-related protein 4
RPH3A: rabphilin
BRIEF: behavior rating inventory of executive function
IQ: intelligence quotient
WASI: Wechsler Abbreviated Scale of Intelligence
TBI: traumatic brain injury
BDNF: brain-derived neurotrophic factor
RBC: red blood cell
NTRK: Neurotrophic Tyrosine Kinase Receptor
SOX: SRY-Box Transcription Factor
CSF: cerebrospinal fluid
TCD: transcranial Doppler
