# Supplementary material for: Proteomic discovery in sickle cell disease: Elevated neurogranin levels in children with sickle cell disease
Source: Proteomics Clin Appl. Author manuscript; Available in PMC 2022 Sep 1. (PMC8666096; doi:10.1002/prca.202100003)
Supplement: Supplemental Data [file NIHMS1757259-supplement-Supplemental_Data.docx]

**Supplemental Data 1:**

There was no significant correlation between initial visit NRGN levels and neuropsychological measures of executive function (Behavior Rating Inventory of Executive Function – BRIEF – Global Executive Composite Score) (rho=0.03, p=0.84) and intelligence quotient (IQ) (Wechsler Abbreviated Scale of Intelligence – WASI – four test score) (rho=0.04, p=0.79). There was no significant correlation between initial vist NRGN levels and lesion number (rho=-0.06, p=0.65) and lesion volume (rho=-0.17, p=0.37).

Using the longitudinal samples from the SIT Trial, NRGN plasma levels from SCD participants with and without SCI did not significantly change over time (coeff -0.0017, 95%CI: -0.009-0.006, p=0.66), over study visits (coeff 0.033, 95%CI: -0.02-0.09, p=0.21), or with increasing age (coeff 0.002, 95%CI: -0.004-0.008, p=0.54) during the SIT Trial. There were no differences in NRGN levels between the observation group and the treatment group over time (coeff -0.003, 95%CI: -0.02-0.01, p=0.69). There was no significant difference in median NRGN levels between the observation group and the treatment group at the initial visit (0.23 vs 0.2 ng/mL, 25-75%IQR: 0.1-0.78 vs. 0.12-0.64 ng/mL, p=0.97) or final visit (0.63 vs 0.56 ng/mL, 25-75%IQR: 0.23-1.85 vs. 0.16-1.9 ng/mL, p=0.95).

Neurocognitive deficits in execution function and memory and academic achievement in children with SCD have been well documented.[80, 81] Though we did not find significant relationships between NRGN and IQ or executive function, more sensitive or specific measures of cognition may be needed to explore these relationships further. Our findings of increased NRGN in children with SCD may be relevant to the etiology of neurocognitive dysfunction that is observed in children affected by the disorder with and without documented brain injury. Further validation studies of larger cohorts with a multi-analyte ELISA of other brain proteins identified in plasma (Table 3) could possibly elucidate links between brain-enriched proteins and neurocognitive outcome. NRGN and other brain protein ELISAs may provide faster, lower cost tests to evaluate whether specific therapies can help prevent brain injury.
